# Ionic Conduction in Lithium Ion Battery Composite Electrode Governs Cross-sectional Reaction Distribution

**DOI:** 10.1038/srep26382

**Published:** 2016-05-19

**Authors:** Yuki Orikasa, Yuma Gogyo, Hisao Yamashige, Misaki Katayama, Kezheng Chen, Takuya Mori, Kentaro Yamamoto, Titus Masese, Yasuhiro Inada, Toshiaki Ohta, Zyun Siroma, Shiro Kato, Hajime Kinoshita, Hajime Arai, Zempachi Ogumi, Yoshiharu Uchimoto

**Affiliations:** 1 Graduate School of Human and Environmental Studies, Kyoto University, Kyoto, 606-8501, Japan; 2 Office of Society-Academia Collaboration for Innovation, Kyoto University, Uji, 611-0011, Japan; 3 Department of Applied Chemistry, Ritsumeikan University, Kusatsu, 525-8577, Japan; 4 Research Organization of Science and Engineering, Ritsumeikan University, Kusatsu, 525-8577, Japan; 5 National Institute of Advanced Industrial Science and Technology, Ikeda, 563-8577, Japan; 6 KRI Inc., Kyoto, 600-8813, Japan

## Abstract

Composite electrodes containing active materials, carbon and binder are widely used in lithium-ion batteries. Since the electrode reaction occurs preferentially in regions with lower resistance, reaction distribution can be happened within composite electrodes. We investigate the relationship between the reaction distribution with depth direction and electronic/ionic conductivity in composite electrodes with changing electrode porosities. Two dimensional X-ray absorption spectroscopy shows that the reaction distribution is happened in lower porosity electrodes. Our developed 6-probe method can measure electronic/ionic conductivity in composite electrodes. The ionic conductivity is decreased for lower porosity electrodes, which governs the reaction distribution of composite electrodes and their performances.

Lithium-ion batteries are promising power sources for electric vehicles, plug-in hybrid electric vehicles and so on^1^. To serve these purposes, it is pivotal to improve various performance attributes of lithium-ion batteries such as capacity, safety, cyclability and high rate capability. The main reaction for lithium-ion batteries is the charge transfer reaction where both lithium ions and electrons react with each other. To improve this reaction site and storage capacity for practical use, composite electrodes comprising active materials, carbon and binder are widely used in lithium-ion batteries as shown Fig. 1. It is well known that their morphology influences the electrochemical performance of batteries. For the improvement of performance in practically used lithium-ion batteries, it is necessary to tune the parameters of composite electrodes such as porosity, compounding ratio and thickness^2^,^3^,^4^,^5^. However, even though many research works have been performed in lithium-ion batteries field, the tuning of composite electrodes has been performed on an empirical basis. This is because determining factors in tuning composite electrodes have not been clearly understood. From a practical standpoint, it is quite essential to scientifically develop the design guide for composite electrodes.

In composite electrodes, electrons and lithium-ions are supplied from current collectors and electrolyte side, respectively shown in Fig. 1. In their conduction paths, there are a number of internal resistances inherent in the composite electrode. The electronic conduction is influenced by metallic and film resistances in aluminum, and carbon and surface resistances of active materials. The ionic conduction is related with ionic resistance in electrolyte and solid state diffusion in active materials. Since the electrode reaction occurs preferentially in regions with lower resistance, reaction distribution might be happened within composite electrodes in depth direction especially in large-scale lithium-ion batteries.

The reaction distribution depends on engineering parameters such as material composition, morphology, porosity in composite electrodes. The morphology of composite electrodes varies the effective electronic and ionic conductivity in composite electrodes^6^,^7^. However the tuning of composite electrodes has been mainly based on the intuition and experiences, since it is difficult to distinguish the electronic conductivity and the ionic conductivity in composite electrodes. For composite electrodes, the traditional 4-probe method cannot be applied because charge-discharge currents are flew during applying voltage.

For the design principle of composite electrodes, it is essential to understand the relationship among battery performance, reaction distribution and electronic/ionic conductivity in composite electrodes. Previously some researchers have studied the distribution model from the theoretical aspect^8^,^9^. There are a few reports made to directly observe the reaction distribution of composite electrodes^10^,^11^,^12^,^13^,^14^,^15^,^16^. More recently, *in situ* observations of reaction distribution have been reported^17^,^18^,^19^. Unfortunately these advanced studies observed distribution phenomena without experimentally addressing the decision factor for the distribution and electrode performance. This study aims to explain their relationship from the experimental results. As the representative engineering parameters, the porosity in composite electrodes is varied. The rate performance, the cross-sectional reaction distribution and electronic/ionic conductivity in various porous composite electrodes are experimentally measured. To investigate the cross-sectional reaction distribution occurring in composite electrodes, two-dimensional (2D) imaging X-ray absorption spectroscopy (XAS) technique is applied^20^. For the measurement of electronic/ionic conductivity in composite electrodes, we have developed a method for simultaneous measurement of electronic and ionic conductivities in composite electrodes. The knowledge is useful to understand the decision factor for charge-discharge performance in lithium-ion batteries and design principle of composite electrodes in any electrochemical devices.

## Results and Discussion

**Figure 2:**
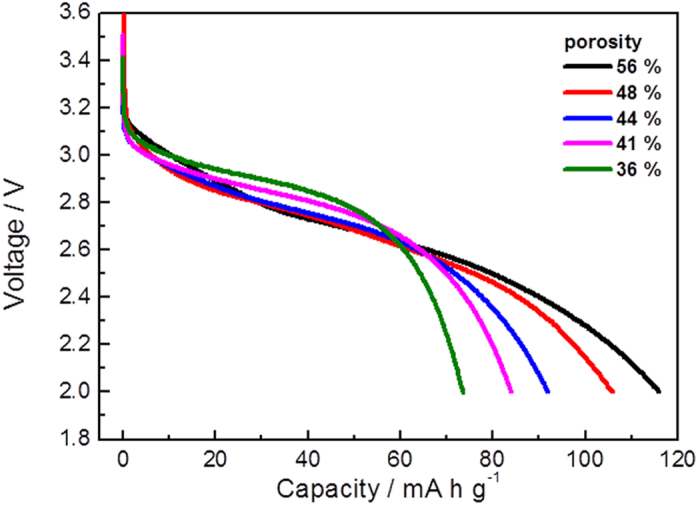
Discharge curves of LiFePO_4_ composite electrodes at 10 C rate at various porosities.

As the representative parameter, the relationship between electrode porosity and discharge capacity at 10 C rate was examined. This study used LiFePO_4_ composite electrodes. Porosity of composite electrodes was varied by changing pressures of a roll press. The cross-sectional SEM images of LiFePO_4_ composite electrodes pressed at various pressures and their porosity are shown in Fig. S1. The difference in porosity, pore size and adhesion exerts influence on the electrochemical characteristics. Discharge curves for LiFePO_4_ electrodes with different porosities at 10 C rate are shown in Fig. 2. The discharge capacity is increased with the increasing porosity from 36% to 56%. A sharp decrease of the voltage at the terminal of the discharge profile is observed for low porosity electrodes (36% and 41%). The equilibrium potential of LiFePO_4_ is about 3.45 V^21^. The voltage drop from the equilibrium potential is mainly termed as ohmic drop, which is influenced by the internal resistance of the battery. A lower discharge voltage demonstrates a large IR drop for the high porosity electrodes (56% and 48%). The porosity of composite electrode impacts on the high rate discharge behavior. In the following study, we investigate the reaction distribution from the current collector to electrode surface and the effective electronic and ionic conductivity in the composite electrodes.

**Figure 3:**
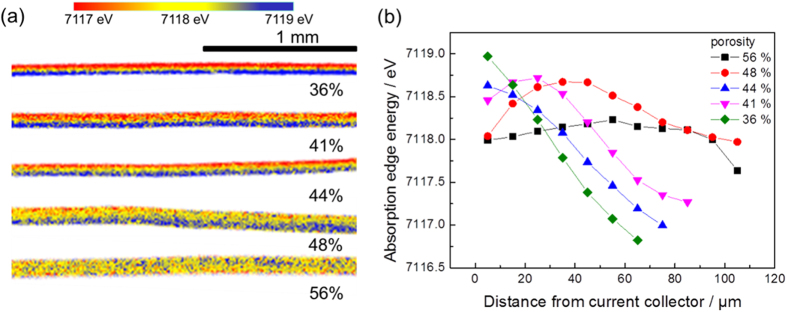
(**a**) Cross section two dimensional mapping of absorption energy at Fe K-edge in LiFePO_4_ composite electrodes. Current collector is located at the bottom side. (**b**) Absorption edge energy as a function of distance from the current collector.

As the LiFePO_4_ electrode is discharged, the absorption energy of the Fe-*K*edge X-ray absorption spectra (XAS) decreases as shown in Fig. S2^22^. By using two-dimensional detector, we can obtain special resolved XAS spectra (Fig. S3). Mapping the absorption energy as a function of pixel position provides the information about cross-sectional reaction distribution of the electrodes^20^. Sample for this mesurement is explained in Fig. S4. Figure 3a shows the cross-sectional reaction distribution mappings of the electrodes with different porosities discharged to the nominal composition of Li_*x*_FePO_4_ approached *x* = 0.45 at 10 C rate. The color changes from blue to red represent that the absorption energy decreases corresponding to the discharge reaction taking place. Therefore, the transition from blue to red in the color bars indicates that the discharge reaction proceeds. Apparently inhomogeneous reaction is caused especially for low porosity electrodes. The absorption edge energy as a function of distance from current collector for various pressed electrodes is plotted in Fig. 3b. When the porosity of the electrode is 44%, 41%, 36%, the reaction occurs preferentially from electrode/electrolyte interface. However, when the porosity of the electrode is 56% or 48%, the uniform reaction is achieved. The reaction distribution in depth direction has been reported in the active materials with mono-phasic reaction such as LiCoO_2_^23^ and LiNi_1/3_Co_1/3_Mn_1/3_O_2_^24^,^25^.

**Figure 4:**
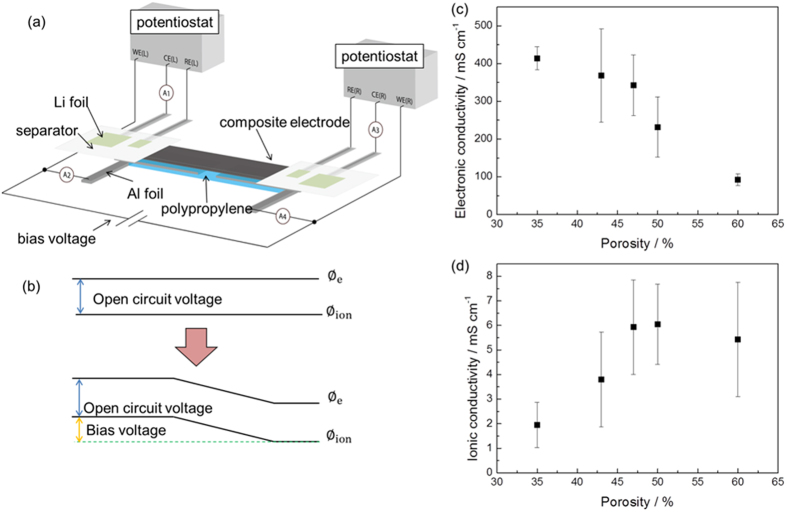
(**a**) Measurement setup for electronic and ionic conductivities measurement in composite electrodes. (**b**) Schematic illustration of the potential profile before and after applying a bias voltage. The potential difference between in the ionic conductor and in the electronic conductor is kept throughout the sample. Effective (**c**) electronic and (**d**) ionic conductivity as a function of porosity in LiFePO_4_ composite electrodes.

The impact of porosity on cross-sectional reaction distribution can be attributed to the effective ionic and electronic conductivities. In order to measure the effective ionic and electronic conductivities, we have developed a method for simultaneous measurement of electronic and ionic conductivities in composite electrodes based on the proposed principle^26^. Figure 4a shows the measurement setup and the arrangement of the electrodes and samples. The electrochemical cell contains 6 probes connecting with the composite electrode. For the electronic and the ionic conduction electrodes, Al foils and Li foils were used, respectively. Sample detail is explained in Fig. S5. Two potentiostats and bias voltage were connected to the cell. After open circuit voltage was measured, the two potentiostats were operated with this voltage as the set point. And then, a bias voltage was applied between the two working electrodes. In this case, the potential though the composite electrode was kept as the setting voltage as shown in Fig. 4b. The ionic current was measured at A1 current meter, and the electronic current was measured at A2 current meter. By using Ohm’s law and the definition of conductivity we can calculate the electronic and ionic conductivity (Fig. S6).

Figure 4c,d shows the effective electronic and ionic conductivities for various porosity composite electrodes. The electronic conductivity is at least 10 times higher than the ionic conductivity, which implies the dominant contribution of the ionic conduction to the electrochemical performance. For the high porosity electrodes, the effective ionic conductivity is almost constant. When the porosities of the electrodes are less than 47%, the ionic conductivity decreased. This result indicates that the effective ionic conductivity is decreased due to the narrow and distorted ion diffusion paths within the composite electrodes. The narrow and distorted pores impede movement of lithium ions and produce a concentration gradient within the composite electrode, consequently leading to large concentration polarization especially in the end of discharge reaction. On the other hand, the electronic conductivity decreases with increasing porosity. The effective electronic conductivity for high porosity electrodes is low due to poor contact of particles, as apparent in the SEM images (Fig. S1). Poor conductive networks cause relatively high ohmic drop in the discharge reaction shown in Fig. 2. The electronic conductivity of LiFePO_4_ is about 5 × 10^−8^ S cm^−1 ^27 which is much lower than the layered oxide, LiCoO_2_ (1 × 10^−3^ S cm^−1^)^28^. However, in the practical applications, LiFePO_4_ of nano-sized particles is used with carbon coating. In this case, the electronic conductivity of LiFePO_4_ itself does not govern the rate capability.

**Figure 5:**
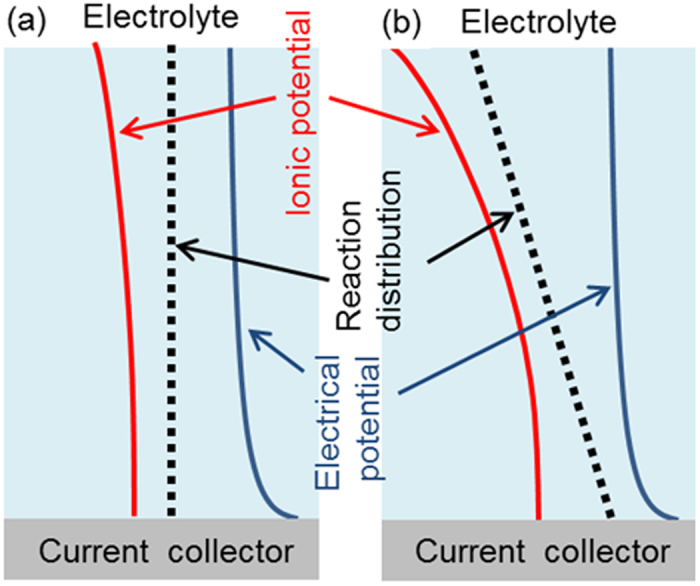
Ionic and electrical potential distribution for (**a**) high porosity electrode and (**b**) low porosity electrode.

The experimental data for the reaction distribution and the effective electronic/ionic conductivity reveals the reaction phenomena in composite electrodes. Figure 5 shows a schematic of ionic and electrical potentials within a composite electrode. Difference between ionic potential and electrical potential is the driving force for the electrode reaction. For low porosity electrodes, ionic potential has a sharp inclination due to a low effective ionic conductivity. As noted above, the low effective ionic conductivity is ascribed to a narrow and distorted pore. On the other hand, electrical potential remain constant at all depths due to a high effective electronic conductivity. As a result, the difference between ionic potential and electrical potential at the surface of the electrode is larger than at the side of current collector. As such, for low porosity electrodes, the reaction occurs preferentially from electrode/electrolyte interface. For high porosity electrodes, the variation of ionic potential is small owing to a high effective ionic conductivity. Large pore size and the favorable path of lithium ion lead to a high effective ionic conductivity. Furthermore, the electrical potential variation is also small because the effective electronic conductivity is enough compared with the ionic conduction. The difference between ionic potential and electrical potential remains constant at all depths in composite electrodes and a uniform reaction is achieved. Therefore, the cross-sectional reaction distribution depends on the effective ionic conductivity and governs the electrochemical properties in composite electrodes. During charge-discharge reaction, the volume change between LiFePO_4_ and FePO_4_ might influence the battery performances especially in highly pressed electrodes. If the volume change governs the rate performance, the reaction distribution in depth direction is not caused, which is not consistent with the reaction distribution observed in the low porosity electrodes.

## Conclusion

We have experimentally investigated the cross-sectional reaction distribution and the effective electronic/ionic conductivity of LiFePO_4_ composite electrodes with various porosities in lithium-ion batteries. Composite electrodes with low porosity cause a large polarization in high rate discharge reaction and decrease their capacity. In these electrodes, the discharge reaction occurs preferentially at the top surface of the electrode near electrolyte side. Further, low porosity results in a low effective ionic conductivity. This study clearly reveals the ionic conduction in the composite electrodes is the governing factor of lithium-ion battery performance. Control of ionic conductivities in composite electrodes is important to further improve the performance of lithium-ion batteries.

## Methods

The active material, carbon-coated LiFePO_4_ powder, with an average particle size of 200 nm was used. The conductive additive, acetylene black, with a mean particle size of 40 nm was acquired from Denka Black (Japan). PolyVinylidene-DiFluoride (PVDF) binder was purchased from Kureha (Japan). For electrode preparation, 75 *wt*% carbon-coated LiFePO_4_ powder, 10 *wt*% acetylene black and 15 *wt*% PVDF were mixed in 1-methyl-2-pyrrolidinone anhydrous (NMP, Wako) solvent. The slurries were coated onto aluminum foil using a doctor blade. Drying was done at 70 °C to remove solvent, and additional drying was performed at 80 °C in a vacuum oven to vaporize the residual solvent. These composite electrodes were densified at 0 kgf, 300 kgf, 600 kgf, 900 kgf, and 1200 kgf pressures to control their porosity.

The LiFePO_4_ electrodes were assembled in electrochemical flat cells with lithium metal (Honjo metal, Japan) as the counter electrode. Two separators (Celgard 2500) were placed between the electrodes. 1M LiPF_6_ in a 3:7 volume ratio of ethylene carbonate (EC) and diethyl carbonate (EMC) was used as the electrolyte solution. The assembly of the cell was carried out in a dry glove box under an argon atmosphere. After charged to 4.2 V at 0.2 C, the discharge properties were measured to 2.0 V at a rate of 10 C at 25 °C.

The cross sections of the composite electrodes were examined using a cross section polisher (CP, JEOL SM-09010) and scanning electron microscope (SEM, Hitachi SU-70). In order to observe the cross section views of the composite electrodes, the cross sections of the composite electrodes were polished by CP and observed by SEM.

The LiFePO_4_ electrodes were discharged under 25 °C at 10 C rate until the nominal composition of Li_*x*_FePO_4_ approached *x* = 0.45. As soon as the electrodes were discharged, they were removed from the cells, rinsed in dimethyl carbonate (DMC) and dried. To observe the cross section view, the dried electrodes were fabricated by CP. 2D-imaging XAFS measurements were performed at the beam line BL-4 at Ritsumeikan SR center (Japan). The beam size was 3 (H) × 4 (W) mm^2^. Fe *K*-edge XAS spectra of the LiFePO_4_ electrodes were collected in transmission mode using a CMOS detector. The spatial resolution was 10 μm.

The effective ionic and electronic conductivity in composite electrodes were measured by the 6-probe method. Two aluminum sheets were connected with polypropylene. The measurement principal is already reported^26^, and the cell setup will be explained in the manuscript.

## Additional Information

**How to cite this article**: Orikasa, Y. *et al.* Ionic Conduction in Lithium Ion Battery Composite Electrode Governs Cross-sectional Reaction Distribution. *Sci. Rep.* **6**, 26382; doi: 10.1038/srep26382 (2016).

## Supplementary Material

Supplementary Information

## Figures and Tables

**Figure 1:**
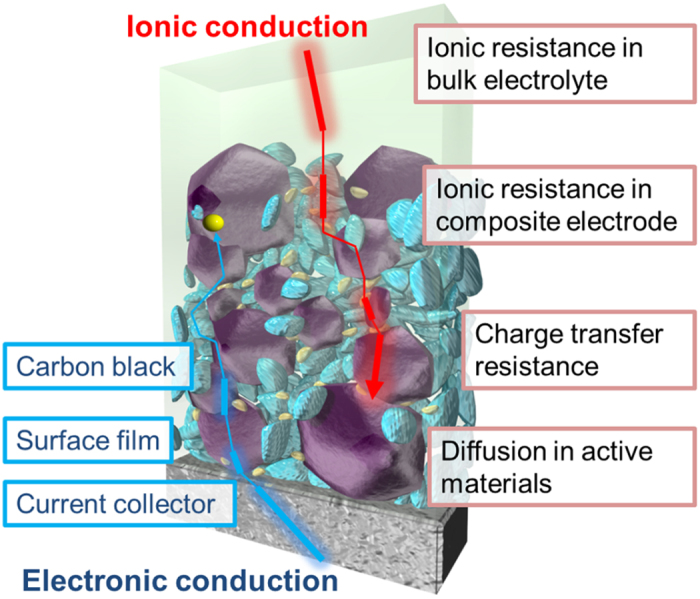
Schematic illustration of a composite electrode in lithium-ion batteries.
