# Supplementary material for: Ionic Conduction in Lithium Ion Battery Composite Electrode Governs Cross-sectional Reaction Distribution
Source: Sci Rep. 2016 May 19;6:26382. doi: 10.1038/srep26382 (PMC4872260; doi:10.1038/srep26382)
Supplement: Supplementary Information [file srep26382-s1.pdf]

Supporting Information

Ionic Conduction in Lithium Ion Battery Composite Electrode Governs Cross-sectional Reaction Distribution

Yuki Orikasa[1], Yuma Gogyo[1], Hisao Yamashige[2], Misaki Katayama[3], Kezheng Chen[1], Takuya Mori[1], Kentaro Yamamoto[1], Titus Masese[1], Yasuhiro Inada[3], Toshiaki Ohta[4], Zyun Siroma[5], Shiro Kato[6], Hajime Kinoshita[6], Hajime Arai[2], Zempachi Ogumi[2] and Yoshiharu Uchimoto[1,*]

[1]Graduate School of Human and Environmental Studies, Kyoto University, Kyoto, 606-8501, JAPAN

[2]Office of Society-Academia Collaboration for Innovation, Kyoto University, Uji, 611-0011, JAPAN

[3]Department of Applied Chemistry, Ritsumeikan University, Kusatsu, 525-8577, JAPAN

[4]Research Organization of Science and Engineering, Ritsumeikan University, Kusatsu, 525-8577, JAPAN

[5]National Institute of Advanced Industrial Science and Technology, Ikeda, 563-8577, JAPAN

[6]KRI Inc., Kyoto, 600-8813, JAPAN

uchimoto.yoshiharu.2n@kyoto-u.ac.jp

S.1 Characterization of $LiFePO_4$ composite electrodes

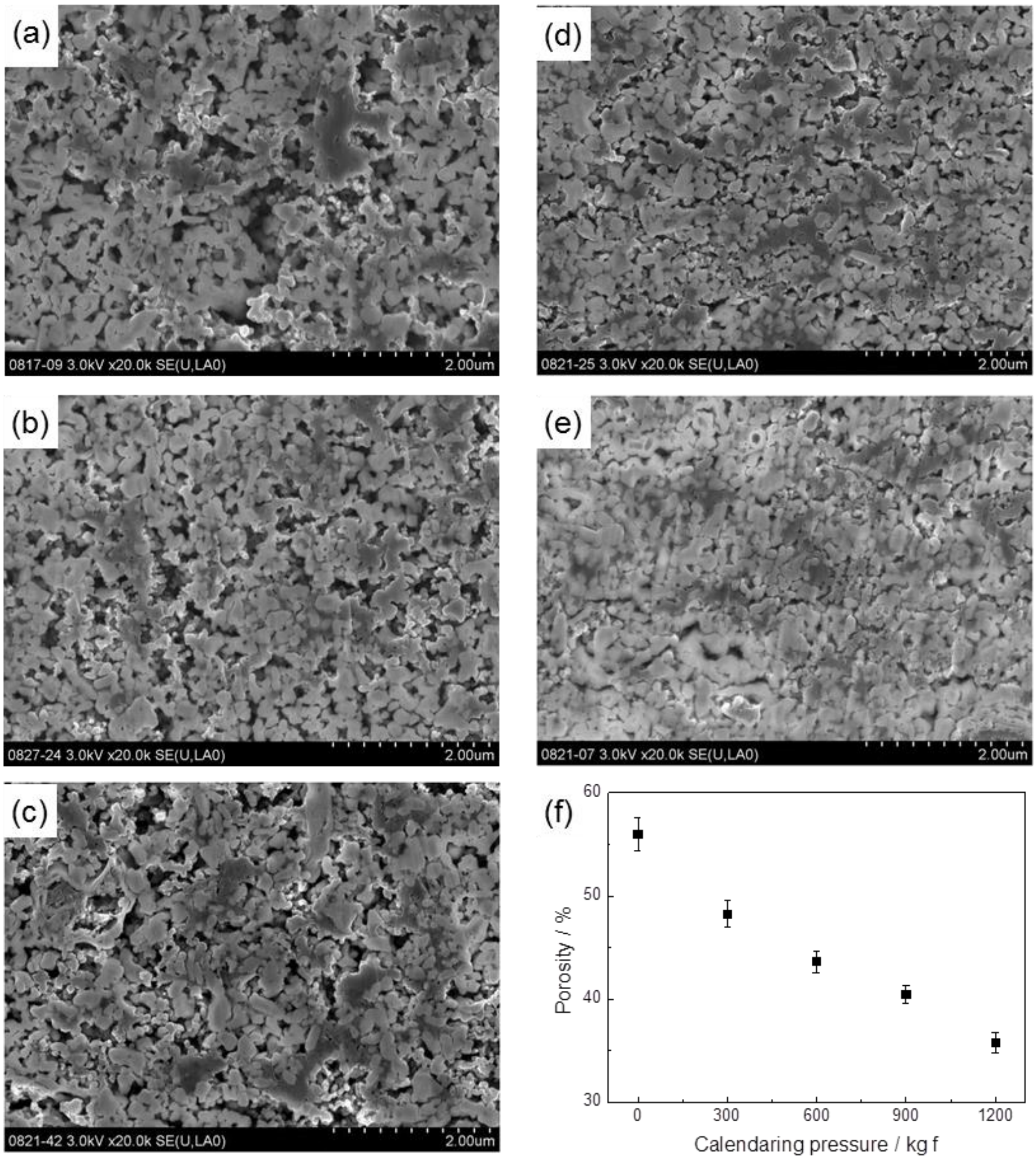

Figure S1. Cross-sectional SEM images of (a) 0 kgf press, (b) 300 kgf press, (c) 600 kgf press, (d) 900 kgf press and (e) 1200 kgf press. (f) Estimated porosity of the composite electrodes as a function of various pressures.

Porosity of composite electrodes is varied by changing pressures of a roll press. The cross-sectional SEM images of $LiFePO_4$ composite electrodes pressed at various pressures are shown in Figs. S1(a) – (e). The active material, the carbon, the binder and pore are distributed. Apparently the porosity decreases with increasing pressures. In addition, the pore size is smaller and adhesion is better with increasing pressure. Pore

size of the electrode with a pressure of 1200 kgf is very small and narrow, with improved adhesion. The porosity, ρ is calculated by using the following equation:

$$\rho = \frac{V_{\mathrm{total}} - V_{\mathrm{dence}}}{V_{\mathrm{total}}} \qquad \text{(S1)},$$

where V_{total} is actual electrode volume (which is electrode area x thickness), V_{dence} is the ideal volume of fully dense electrode. As shown in Fig. S1(f) the estimated porosity decreases with increasing pressure.

S.2 Detection method of overall lithium contents of Li_xFePO_4 in composite electrodes using energy shift of X-ray absorption spectroscopy

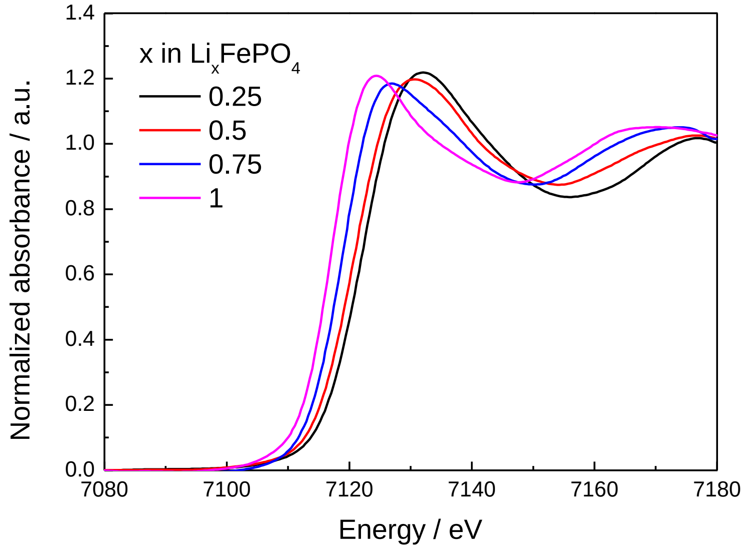

Figure S2. Fe K-edge X-ray absorption spectra of Li_xFePO_4 composite electrodes.

The X-ray absorption edge energy shifts towards higher energy during Li insertion in Li_xFePO_4. In this study, the inflection point of the edge is defined as the absorption edge which reflects the lithium contents in Li_xFePO_4.

S3. Two-dimensional X-ray absorption measurement

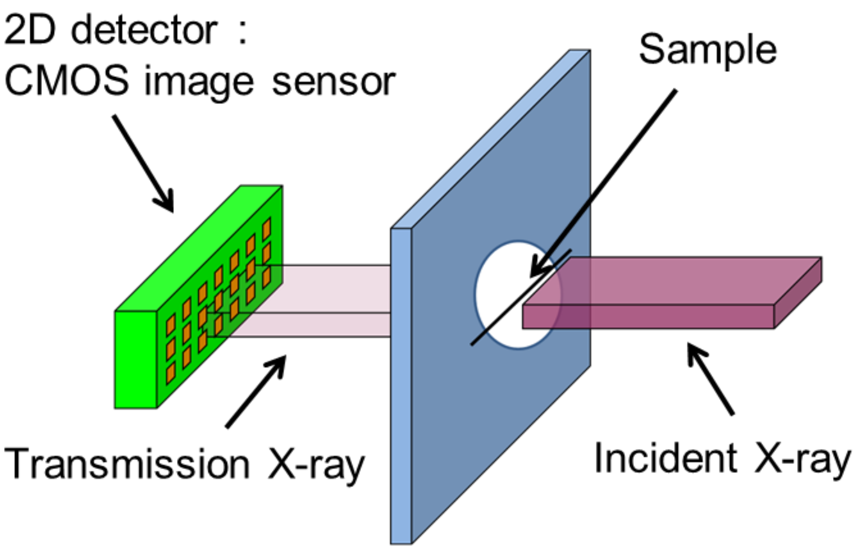

Figure S3. Schematic illustration of measurement setup of two-dimensional X-ray absorption spectroscopy.

The two-dimensional X-ray absorption measurements were performed at the beam line BL-4 in Ritsumeikan SR center (Japan). The cross sectional sample was set in X-ray beam path. The beam size was 3 (H) ×4 (W) mm^2. Fe *K*-edge XAS spectra of the $LiFePO_4$ electrodes were collected in transmission mode. Two-dimensional X-ray detector (Flash2.8, Hamamatsu Photonics) consisting a complementary metal oxide semiconductor (CMOS) image sensor was used.

S.4 Sample preparation of $LiFePO_4$ composite electrodes for two-dimensional X-ray absorption spectroscopy

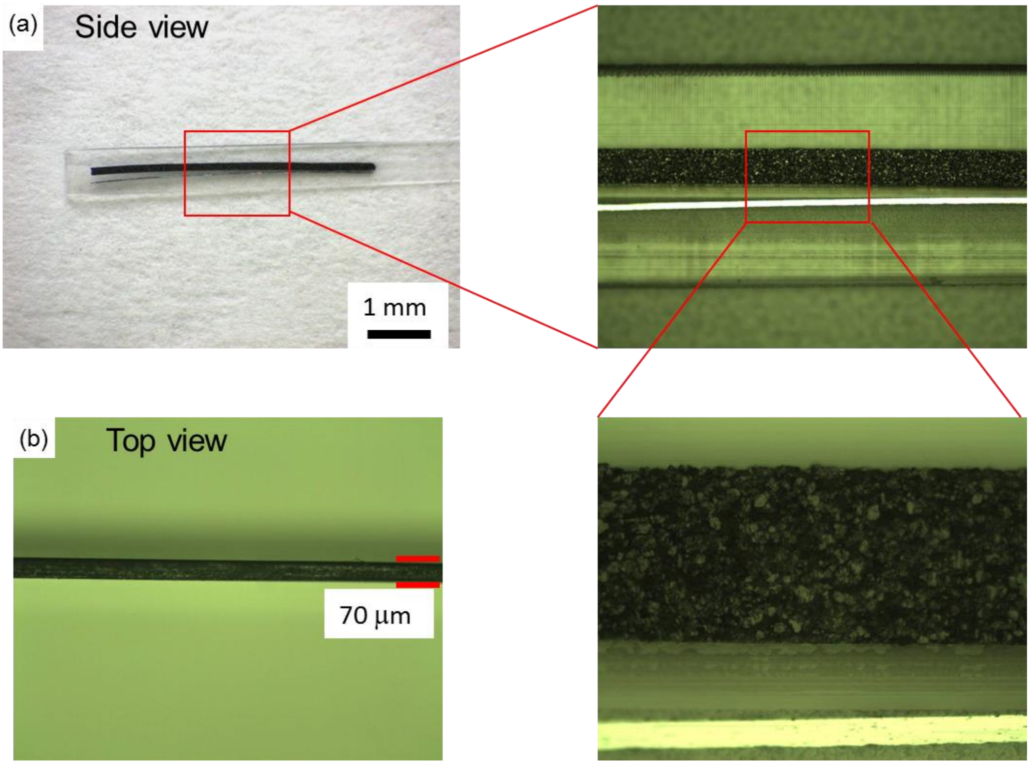

Figure S4. Optical micrograph of composite electrode of (a) side view and (b) top view after cross section polishing.

The $LiFePO_4$ electrodes were discharged under 25°C at 10 C rate until the nominal composition of Li_xFePO_4 approached x = 0.45. As soon as the electrodes were discharged, they were removed from the cells, rinsed in dimethyl carbonate (DMC) and dried. The electrodes were mounted by epoxy resin and the cross-section was prepared using JEOL SM-09010 cross section polisher (CP) (Fig. S2(a)). For X-ray absorption measurement in a transmission mode, sample thickness with X-ray penetration path was set as 70 µm as shown in Fig. S2(b).

S.5 Simultaneous measurements of ionic and electronic conductivity in composite electrodes

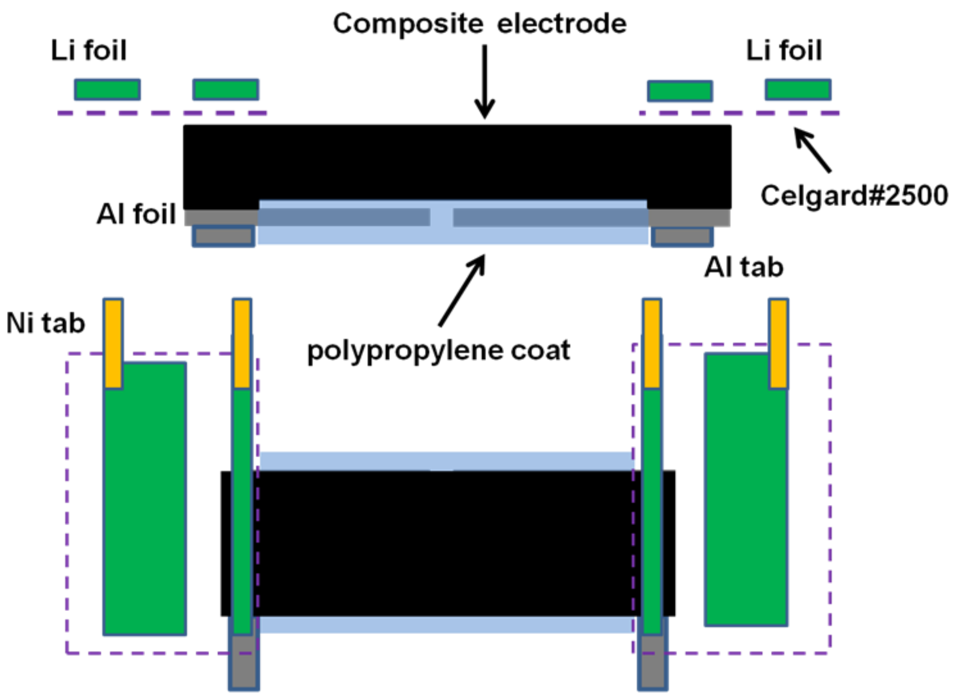

Figure S5. Schematic illustration of (a) cross-sectional view and (b) overhead view of simultaneous measurements of ionic and electronic conductivity in composite electrodes.

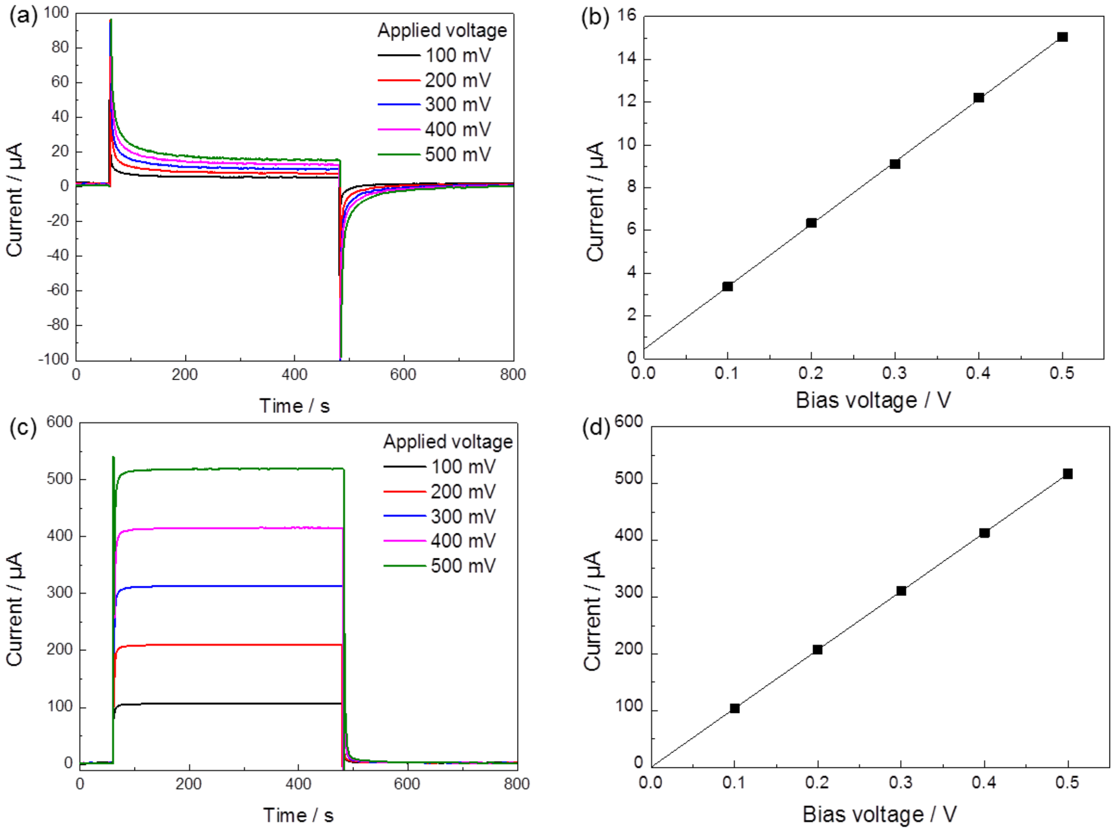

Figuer S6. Representative raw data for ionic and electronic conductivity measurements. (a) time courses of the ionic current, (b) ionic current as a function of bias voltage, (c) time courses of the electronic current, and (d) electronic current as a function of bias voltage in $LiFePO_4$ composite electrodes pressed with 300 kgf.

The two potentiostats were operated with 3.2 V as the set point. And then, different bias voltages (0.1 V, 0.2 V, 0.3 V, 0.4 V, and 0.5 V) was applied between the two working electrodes. The ionic and the electronic currents were measured at around 450 s as shown in Fig. S6 (a) and S6 (c). Using the plot of the current vs. bias voltage, the slope of the least squares line was calculated, which corresponds to the effective ionic / electronic resistance, *R*. The effective ionic / electronic conductivity, σ were calculated by the following equation:

$\sigma = l / (S \times R)$ (S2),

where *l* and *S* mean the length of electrode and the cross sectional area of electrode, respectively.
